# Exploring self-presentation posts of people with depression: themes, stigma, and identity construction

**DOI:** 10.3389/fpubh.2025.1558197

**Published:** 2025-06-10

**Authors:** Lei Gu, Xia Gao

**Affiliations:** ^1^Speech-Language-Hearing Center, School of Foreign Languages, Shanghai Jiao Tong University, Shanghai, China; ^2^The Engineering & Technical College of Chengdu University of Technology, Leshan, China

**Keywords:** social media, mental health communication, identity construction, stigma, cognitive biases

## Abstract

**Introduction:**

Social media plays an increasingly influential role in shaping mental health communication. However, individual expressions related to self-presentation, particularly among people with depression, remain relatively understudied. This study aims to examine how individuals with depression construct and present their identities on Xiaohongshu (RED), a prominent Chinese social media platform.

**Methods:**

We employed semantic network analysis and content analysis to investigate depression-related self-presentation narratives on Xiaohongshu. User posts were systematically analyzed to identify key themes and patterns in identity construction.

**Results:**

Three primary themes driving self-presentation narratives were identified: Mental Health & Treatment, Life Stress & Emotion Management, and Self-awareness & Emotional Experience. These themes are intertwined with experiences of stigma and personal challenges, fostering a narrative shift toward optimism and resilience. This shift supports the formation of three distinct identity types: the Depressed Self, the Optimistic Self, and the Resilient Self. Cognitive biases such as negative filtering, overgeneralization, and catastrophizing were found to influence identity formation.

**Discussion:**

The findings reveal the complex interplay between thematic narratives, cognitive biases, and stigma in online self-presentation among individuals with depression. The study highlights the pivotal role of social media in redefining mental health narratives and shaping online identity construction.

## Introduction

Depression is increasingly common worldwide. According to the World Health Organization, approximately 5% of adults globally suffer from depression (1). This prevalent condition carries serious repercussions, leading individuals to conduct self-harm or even commit suicide (2). In this case, research on depression communication is crucial. Prior studies on depression communication [e.g., (3, 4)] have nevertheless predominantly concentrated on conventional media channels like newspapers and television. Social media, on the other hand, not only provides efficient and accessible platforms for exchanging real-time insights into individuals’ mental states but also offers public opinions on mental disorders and disseminates treatment resources. While there is abundant research on public opinions toward depression, there is a noticeable lack of studies exploring the self-presentation of depression (5, 6). In fact, actively listening to the voices of individuals living with depression not only enriches our understanding of the condition’s complexity, but also provides insights into how those with depression construct and negotiate their identities through storytelling. However, much less is known about how individuals with depression present themselves and construct their identities through first-person narratives on social media—especially within non-Western contexts like China.

This study addresses this gap by examining self-presentation posts on Xiaohongshu to explore how individuals with depression construct their identities through personal narratives, highlighting the role of stigma, cognitive biases, and cultural context in shaping these expressions. Specifically, our study analyzed 40,206 Chinese characters from 115 self-presentation posts by individuals with depression on Xiaohongshu, a prominent Chinese social media platform. The results identified three key themes and highlighted the use of stigma and challenge cues in these posts, which facilitate identity construction influenced by cognitive biases. Our findings highlight how social media supports individuals in expressing and redefining their mental health experiences online.

### Mental health communication on social media

Recent research highlights a shift in mental health communication from traditional media to social media platforms, driven by the democratization of access to information and the ability to connect with others regardless of geographical limits. For example, Gu and Ding (5) conducted a bibliometric analysis over two decades (2002–2022) and found a significant transition from traditional outlets like television and newspapers to social media platforms such as Twitter and Sina Weibo. This shift has, nevertheless, mixed impacts. On the positive side, it facilitates advocacy through personal stories of coping with mental disorders and sharing treatment resources, fostering community support and reducing isolation (7). On the negative side, however, it can reinforce stereotypes, expose individuals to cyberbullying and stigma, and promote unrealistic ideals, worsening symptoms for those with mental health conditions (8).

While existing studies have provided insights into the general trends and varied impacts of social media on mental health communication, they have nonetheless not yet thoroughly explored how individuals with depression specifically present their experiences (9). Addressing this gap is crucial for understanding self-presentation and identity construction, aiding mental health professionals in developing effective support strategies, reducing stigma, and promoting positive narratives (10, 11). It also contributes to discussions about the role of digital platforms in mental health communication, emphasizing the need for responsible media practices (12).

### Stigma communication in mental health

Among the various factors influencing discussions about mental health communication, stigma profoundly shapes how mental health is perceived and discussed online. By definition, stigma refers to the process by which certain characteristics or conditions, such as mental illness, are socially discredited through mechanisms like labeling, stereotyping, separation, status loss, and discrimination (13, 14). This process often results in psychological distress and restricted access to resources for the stigmatized individual. Labeling, in particular, plays a foundational role by assigning individuals to socially constructed categories that may carry negative connotations, thereby triggering further stigma-related processes. While this construct manifests as structural stigma (institutional biases embedded in policies and practices), public stigma (negative attitudes and beliefs held by the general public), and self-stigma (internalized negative attitudes and beliefs) (15), empirical research on mental health communication has predominantly focused on structural and public stigma, frequently neglecting the aspect of self-stigma (5, 6). This knowledge gap highlights the need for more comprehensive empirical research on how people suffering from depression present themselves on social media platforms.

Additionally, stigma communication of mental health is not one-sided, focusing solely on stigma itself, but considers the counteracting power of challenging information. In fact, societal views on mental disorders are moderated by both **stigma cues** and **challenge cues**. In this study, we define stigma cues as communicative elements that reinforce negative perceptions of mental illness, such as labeling, blaming, and viewing individuals as dangerous. These cues contribute to the marginalization and internalized shame experienced by those with mental disorders. Conversely, challenge cues are communicative elements that counteract stigma by promoting recovery, empowerment, and social inclusion. They include expressions of optimism, hope, resilience, and advocacy for mental health awareness (16, 17).

Specifically, stigma cues include labeling (assigning negative stereotypes), blaming (attributing fault to individuals), and perceptions of peril (viewing individuals as dangerous) (16). On the other hand, challenge cues involve optimism (maintaining a positive outlook), hope (emphasizing recovery), social inclusion (promoting connectedness), personification (humanizing the condition to reduce self-blame), and seeing the fight against the disorder as a winnable battle (17). Several studies have provided empirical evidence on how these cues are reflected in the communication of mental disorders (18–22). For example, Corrigan et al. (18) found that when the general public believe that people with mental disorders can control their conditions (controllability of mental disorders), they are more likely to blame the depressed individuals for their conditions (the blame cue). In contrast, Yanos et al. (22) found that nurturing a hopeful perspective (the hope cue) toward one’s mental disorder fosters a positive “illness identity,” significantly shaping how individuals perceive and engage with their condition.

These empirical studies altogether suggest that addressing stigma cues and promoting challenge cues are crucial for creating a supportive environment. However, the extant research lacks specificity on how the depressed individuals navigate these cues in their self-presentations on social media. Gaining a deeper understanding of these nuances can provide valuable insights into the interplay between mental health stigma and identity construction online. This knowledge can, in turn, guide mental health professionals and advocates in better supporting individuals with depression in the digital age.

### Narrative identity construction through cognitive biases

Building on the understanding of stigma communication in mental health, it is crucial to explore how this concept intertwines with cognitive processes in shaping the ways people with mental disorders perceive their experiences and navigate their mental health journeys. Past research often employs a reductionist approach, interpreting mental disorders primarily through biological or physiological lenses (23). This approach may overlook psychological elements like **cognitive biases**, which significantly influence self-perception and interactions (24). For instance, biases such as **negative filtering** (focusing only on negatives), **overgeneralization** (drawing broad negative conclusions from a single event), and **catastrophizing** (expecting the worst outcome) profoundly impact how individuals with social anxiety construct their identities on social media (25–27).

In addition, individuals with mental disorders frequently use social media to construct narratives influenced by cognitive biases, helping them understand and navigate their identities (28). These narratives, reflecting lived experiences, offer insights into digital expression and depression management. The “**Depressed Self**” narrative, laden with negative filtering and catastrophizing, often highlights personal struggles and distorted perceptions (29, 30). In contrast, the “**Optimistic Self**” presents resilience and moments of positivity, challenging traditional depressive narratives through cognitive restructuring (31, 32). Meanwhile, the “**Resilient Self**” emphasizes overcoming adversity and embracing self-care despite ongoing challenges (33). For instance, Bathina et al. (29) analyzed the social media language of individuals with depression, finding a significantly higher frequency of cognitive distortions like overgeneralization and personalizing. These patterns illustrate the self-referential and negatively biased thinking typical of the Depressed Self, highlighting how social media content provides rich data that lends itself well to the identification and analysis of such cognitive biases by researchers. However, existing research does not sufficiently explore how individuals actively reshape their identities in response to stigma communication in mental health, pointing to an important area for future studies.

### Self-presentation about depression on social media

Past research into self-presentation of depression on social media have explored prevalent themes and associated stigma and challenge cues. First of all, past studies have identified recurring themes in online depression discussions, particularly focusing on the negative experiences individuals have encountered. For example, Lachmar et al. (34) analyzed Twitter discussions using #MyDepressionLooksLike, identifying seven negative themes: dysfunctional thoughts, lifestyle challenges, social struggles, hiding behind a mask, apathy and sadness, suicidal thoughts and behaviors, and seeking relief. Conversely, past studies have also highlighted themes related to companionship, coping skills, and emotional support. For instance, Sun and Fichman (35) examined an online depression self-help group in China, highlighting themes like timely intervention and empathy in therapy. Similarly, Sik et al. (36) found discussion topics in an online group that included companionship support, daily communication, and emotional support. The diversity of themes from these studies highlights the varied and complex ways individuals experience and communicate about depression on social media.

Regarding stigma and challenge cues, research has examined various platforms to understand these dynamics. For example, Li et al. (9) studied autobiographical videos on Douyin and found a mix of positive, neutral, and negative sentiments, particularly among female creators, with most posts highlighting challenge cues over stigma. Additionally, Gaus et al. (37) analyzed YouTube depression personal account videos, particularly those by youth, highlighting references to youth-related issues, suicidality, and self-harm. Furthermore, Moore et al. (38) investigated stigma and disclosure in perinatal depression discussions on an online forum, finding internal and external barriers but also promoting help-seeking behavior. These studies collectively found a mix of challenge and stigma cues in online content about depression.

Unlike Western countries where cases of self-presentation depression have been frequently reported, fewer studies on depression self-presentation have been done in China, where stigma and undertreatment are prevalent (39). The lack of reported cases in China might contribute to social fear, cultural norms emphasizing “saving face,” and individual attributions to internal factors like overthinking or vulnerability (8, 40, 41). For studies that investigated Chinese online users’ self-presentation of depression, they primarily uncovered how people with depression made sense of their experiences (e.g., complaining, regret, superiority, and discovery) (28), as well as coping strategies used to combat depressive disorders (23). Given the limited coverage of self-presentation of depression on social media in China, we do not know much about the topics people with depression address online, their self-presentations, and notably, their strategies for negotiating their identities amidst stigma and challenge cues. China’s vast social media user base serves as an optimal environment for studying how individuals with depression present themselves online. In light of this, the present study aims to address this gap by examining how people with depression make sense of their mental health condition through self-presentation narratives shared on online platforms.

### Research questions

In response to the research gaps and conflicts identified in the current literature—specifically, the limited exploration of how individuals with depression present their experiences on Chinese social media, the nuances in stigma and challenge cues within these presentations, and the impact of these narratives on identity construction—this study asks three specific questions.

RQ1:

What are the prevailing themes evident in self-presentation social media posts about depression, laying the foundation for understanding the content landscape?

RQ2:

How do stigma and challenge cues manifest within self-presentation social media posts about depression, offering insights into the nuanced experiences shared by users?

RQ3:

How do people with depression construct and negotiate their identity through stigma and challenge cues—and how do cognitive biases influence this identity construction—within the context of their mental health struggles, providing a richer understanding of their diverse and nuanced selves?

## Methods

### Data source

Xiaohongshu, also known as Little Red Book or RED, is an ideal platform for exploring depression from a first-person perspective. RED is one of China’s largest user-generated content communities (42). It boasts an extensive user base that primarily consists of young individuals, who are particularly active in discussing a wide range of topics, including personal mental health challenges (43). This demographic trend is crucial as younger users are generally more open to sharing and engaging with content related to mental health issues, including detailed personal experiences with depression. Additionally, the platform’s sophisticated recommendation system tailors content discovery to individual preferences, effectively surfacing relevant posts about depression to users interested in similar topics, thus enhancing the visibility of these discussions (42). Lastly, RED’s privacy settings allow users to discuss sensitive topics like depression while maintaining a level of anonymity, promoting a safe environment for open and honest dialog.

### Data sampling

To gather relevant data, this study employed a systematic sampling method involving predetermined keywords, meta-data extraction through web crawlers, and manual screening of posts. Initially, this study utilized a variety of hashtags such as #depression, #depressive disorder, #major depression, #mild depression, and #antidepressant, among others, to identify all potential posts. While these tags may not cover every relevant post, they proved effective after several iterations of searching. Subsequently, this study developed Python scripts to automate the process of identifying posts containing these tags on RED. This script enabled us to collect essential information which was categorized into three distinct groups: (1) post titles and contents, (2) engagement metrics (such as likes, saves, and comments), and (3) other meta-data (including post ID, URL, timestamp, and location). Because of the constraints of the RED algorithm, each search was restricted to approximately 200 results. To augment the sample size, this search encompassed the period from January 1, 2024, to January 7, 2024, yielding over 2,000 post entries.

Following data collection, each post was individually reviewed to ensure its relevance and authenticity for the study. Only posts containing static graphics were included, while video content was excluded in order to maintain consistency and avoid potential confounding effects from multimodal information. The dataset was limited to self-presentational accounts of depression, excluding educational posts by medical professionals, third-person narratives, and content related to fictional media such as films or television shows. To assess authenticity, we used a multi-step screening process. User profiles were manually examined for recurring references to depression, including mentions of therapy, medication, or emotional struggles. Posts that appeared to serve commercial purposes, such as those featuring product placements, advertising hashtags, or branded language, were excluded. We also evaluated the narrative and visual elements of each post for signs of personal experience and emotional sincerity. For instance, posts that described specific life challenges in a reflective tone and included casual, personal imagery were considered authentic, whereas those relying on stock images or generic motivational quotes without personal context were excluded. Following the manual review process outlined earlier, this study identified 115 viable posts, spanning publication dates from July 2020 to January 2024.

### Data preprocessing and coding

As the answers to the research questions relied on the content of the posts, this study only analyzed the titles and content of the posts. This study initially found diverse elements in post titles and contents, such as Chinese characters, English words, emoticons, numbers, hashtags, and special characters, and finally excluded non-Chinese characters which were not relevant to this study, resulting in a total length of refined Chinese text strings of 40,206 characters (mean = 320.83, SD = 248.86). This study then used the Jieba Python library to refine the text data, tokenizing words, removing common stopwords, and integrating synonyms into the thesaurus files for substitution. This study used a selective filtering approach for nouns, verbs, adjectives, and adverbs, concentrating on semantically significant elements of the text essential for identifying prevailing themes in self-presentation social media posts. This method was chosen to handle the large data volume while preserving the depth needed to capture the nuanced experiences of individuals with depression. By focusing on these key word types, we maintained the essence and complexity of the users’ layered narratives. Furthermore, this study identified the top 100 words based on frequency and constructed a co-occurrence matrix based on the the words for latter analysis.

Second, this study delved deep to examine the stigma and challenge cues within each post, particularly focusing on those with predominantly positive and negative tones. Based on established research [cf. (9, 44, 45)], this study adopted six *a priori* coding categories for stigma and challenge cues. In essence, stigma cues involve **negative attribution** (attributing depression to personal failings, like “I blame myself for my depression”), **peril** (depression leads to suicide, self-harm, or burdening others, like “I do not want to live anymore due to my depression,” “my family become disgraced because of my condition”), and **status loss** (the perceived or actual decline in one’s social standing, interpersonal relationships, or role functioning due to depression, like “I have lost friends and social connections because of my depression”). On the other hand, challenge cues consist of **hope** (maintaining a positive outlook on overcoming depression, like “I believe things will get better for me”), **resilience** (individual determined efforts to overcome depression, like “I am capable of coping despite my challenges”), and **advocacy** (strategic actions and efforts aimed at promoting awareness, influencing policy, and fighting against stigma and discrimination related to depression, like “I participate in mental health advocacy groups to promote understanding, fight stigma, and push for better mental health policies and support systems”). The coding job for stigma cues and challenge cues was done by two Chinese graduate students who initially coded all posts independently and then discussed any discrepancies. The inter-rater reliability demonstrated high consistency, with Krippendorff’s alpha exceeding 0.9 for all coding variables.

To further clarify the analytical focus, the data preprocessing and coding procedures described above correspond to the study’s three research questions. Specifically, the text processing and semantic network construction were conducted to address RQ1, which explores prevailing themes in self-presentation posts. The identification and coding of stigma and challenge cues were designed to answer RQ2, which investigates how these cues manifest in user narratives. Finally, RQ3 was addressed by examining how the presence and combination of these cues contributed to identity construction and reflected underlying cognitive biases.

### Data analysis

For RQ1, this study decided to use semantic network analysis (SNA) instead of topic modeling (TM). This choice was made because (1) TM struggles with short texts and capturing word associations (46); (2) Most posts have a mix of themes (multiple member issues), making it difficult to assign themes at the post level. On the contrary, SNA, especially when employed with tools like VOSviewer (47), allows for a deeper exploration of textual data within social networks, uncovering hidden patterns and relationships. By assessing association strength, this study created a visual representation of a semantic network to gauge the proximity of items in the dataset. This study evaluated this network using VOSviewer’s Link (L) and Total Link Strength (TLS) measures, where L tallies occurrences between items and TLS aggregates the strength of connections associated with specific items in the network [cf. (48, 49)]. It should note that this study used Chinese text for the semantic network to preserve original meanings, while also providing an English translation of keywords for wider accessibility.

For RQ2, this study conducted a content analysis of stigma (negative contribution, peril, status loss) and challenge (hope, resilience, and advocacy) cues to delve into the experiences of individuals with depression. For RQ3, this study adopted a functional perspective and employed inductive content analysis to explore how individuals construct their identities concerning depression. While the coding of stigma and challenge cues was deductively guided by prior literature (RQ2), the identity types (Depressed Self, Optimistic Self, and Resilient Self) emerged inductively from patterns in how users framed their mental health experiences in relation to those cues. Definitions of these identity types were refined through iterative team discussions and grounded in narrative identity theory [e.g., (22)]. Additionally, cognitive biases were defined based on established psychological frameworks [e.g., (25)], with a focus on negative filtering (emphasizing only negative aspects), overgeneralization (drawing broad negative conclusions from limited events), and catastrophizing (anticipating worst-case outcomes). These were identified through linguistic indicators and narrative patterns expressing distorted thinking. Given the interpretive nature of this analysis, coding was carried out by trained coders who collaboratively reviewed and discussed examples to ensure consistency and transparency.

## Results

### RQ1

**Figure 1:**
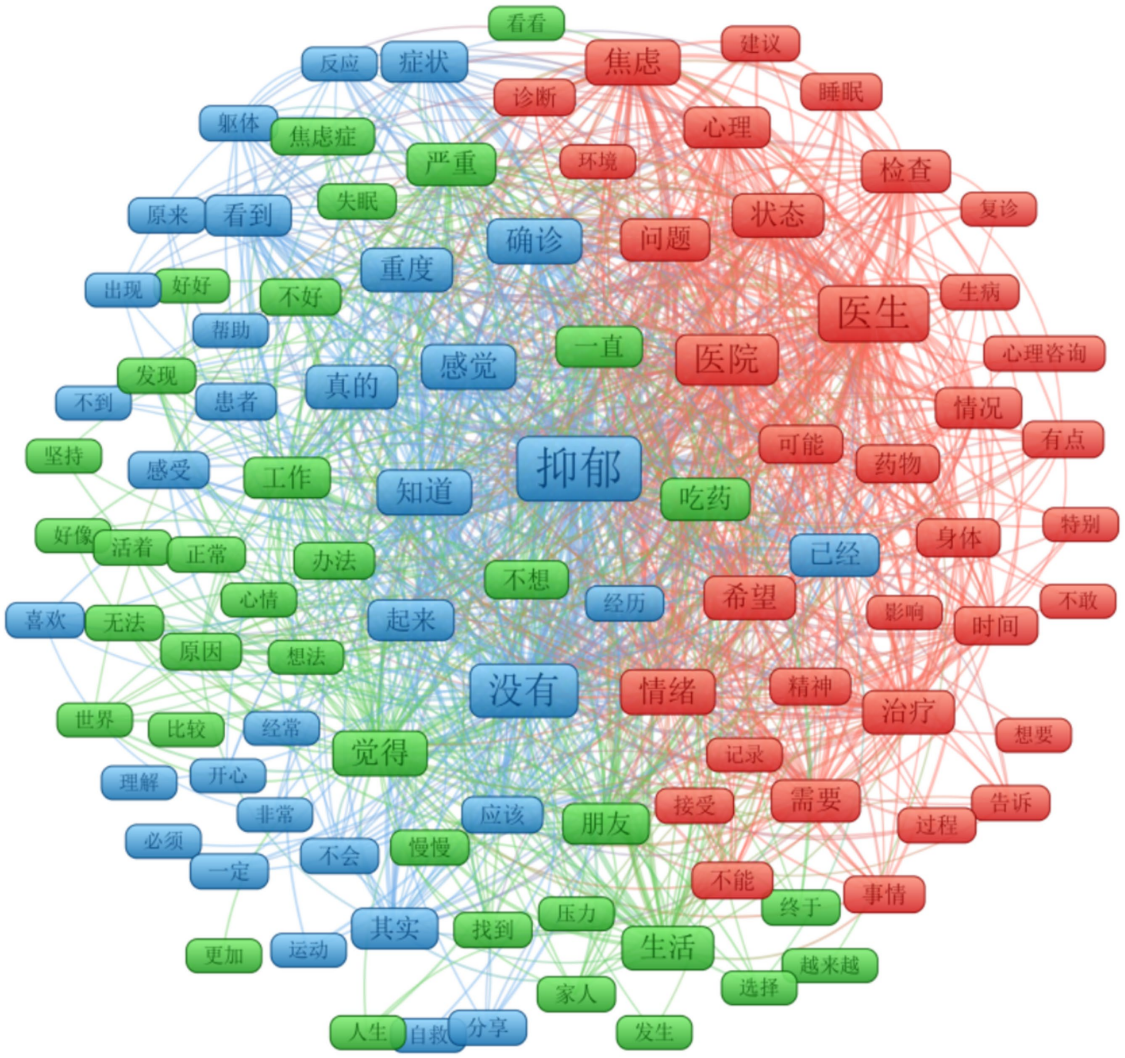
Semantic network analysis of keywords from posts.

Research Question 1 centered on identifying predominant themes present in self-presentation social media posts discussing depression. As shown in Figure 1 (refer to Table S1 in the supplementary materials for the English Translation), the keywords can be divided into three clusters (L = 4,991, LTS = 2,3,842). These clusters encompass (1) **Mental Health and Treatment** (depicted in red, *N* = 35), (2) **Life Stress and Emotion Management** (represented in green, with *N* = 35), and (3) **Self-Awareness and Emotional Experience** (shown in blue, *N* = 34). Firstly, Mental Health and Treatment encompasses seeking assistance from doctors and hospitals, undergoing diagnosis and treatment, managing anxiety and other mental health issues, as well as addressing sleep-related challenges in the context of depression and recovery processes. Secondly, Life Stress and Emotion Management focuses on managing life stressors and emotions, including adopting coping strategies, seeking support from family and friends, maintaining mental well-being amidst challenges, and striving for a sense of normalcy in daily life. Thirdly, Self-Awareness and Emotional Experience explores self-awareness and emotional experiences, including accepting one’s feelings and emotions, understanding symptoms of conditions like anxiety and depression, seeking help and support, and engaging in self-care practices and activities to enhance overall well-being. It should note that Life Stress and Emotion Management (green cluster) and Self-Awareness and Emotional Experience (blue cluster) tend to overlap somewhat, possibly due to their shared focus on emotion-related issues stemming from depression. In summary, it is important to note that, in addition to depression experiences and coping strategies (which have been widely studied in previous studies), the uncovered themes of self-awareness and emotional experiences reveal the inner feelings of individuals suffering from depression, which have been relatively underreported.

### RQ2

Research Question 2 delved into the stigma and challenge indicators present in self-presentation posts authored by individuals with depression. Overall, three key findings emerge: (1) There is a notable prevalence of stigma cues (*N* = 164) over challenge cues (*N* = 110) in the posts; (2) A single post may exhibit a combination of cues, as creators frequently interweave within posts their personal experiences (typically negative), treatment encounters (typically negative), attempts at mitigation (typically positive), and aspirations for recovery (typically positive); (3) Despite the mixed cues within the body of the post, the narrative tends to skew toward expressions of relief and hope, particularly in the latter portion.

Of the stigma cues identified, status loss was utilized most frequently (*N* = 126), followed by peril (*N* = 32) and negative attribution (*N* = 6). Conversely, among the challenge cues, hope was the most prevalent (*N* = 45), followed by resilience (*N* = 38) and advocacy (*N* = 6). The descriptive statistics for the stigma and challenge cues were summarized in Table 1.

**Table 1:** Descriptive statistics for stigma and challenge cues.

Cue type	Subcategory	N(%)	Krippendorff’s alpha
Stigma	Negative attribution	6(3.66)	0.95
	Peril	32(19.51)	0.96
	Status loss	126(76.83)	0.94
Challenge	Hope	45(40.91)	0.93
	Resilience	59(53.64)	0.95
	Advocacy	6(5.45)	0.94

“Krippendorff’s alpha” measures the inter-rater reliability of coded variables.

### RQ3

Research Question 3 focuses on how people with depression construct and negotiate their identities (**the Depressed Self**, **the Optimistic Self**, and **the Resilient Self**) through stigma and challenge cues (**negative attribution**, **peril**, **status loss**, **hope**, **resilience**, and **advocacy**). Cognitive biases, such as **negative filtering**, **overgeneralization**, and **catastrophizing**, significantly influence these identity constructions and are evident in how individuals narrate their experiences on social media.

#### The depressed self

The Depressed Self is constructed through negative attribution cues, peril cues, and status loss cues. Firstly, negative attribution cues are marked by self-blame, remorse, and emotional turmoil, heavily influenced by cognitive biases like negative filtering and catastrophizing, as individuals often attribute their struggles to personal failings, internalizing blame and expressing deep remorse. For example, one said, “***My situation is self-inflicted, and no one needs to take responsibility for my psychological issues. Every day off track is a loss for me, and such incidents will be a stain on my career***.” These statements reflect a profound internalization of guilt and responsibility for their mental state, highlighting feelings of worthlessness and shame. The cognitive bias of negative filtering distorts their perception, causing them to focus solely on their failures and amplify their emotional turmoil. Additionally, catastrophizing is also evident as the individual anticipates the worst outcomes, seeing each deviation as a significant loss and a permanent stain on their career, thus intensifying their sense of despair and hopelessness.

Secondly, the Depressed Self is also constructed by peril cues, which reveal personal struggles with challenges like suicidal ideation and strained relationships. Cognitive biases such as overgeneralization and catastrophizing exacerbate these perceptions. For example, an individual posted, “***When my depression gets bad, I feel like there’s no escape. I’ve lost friends because of this, and it feels like no one will ever understand or help me. Every day is a battle just to stay safe***.” Such statements underscore the intense, often hidden struggles individuals face in maintaining safety and stability. Overgeneralization leads them to draw broad negative conclusions from these experiences, believing that losing a few friends means they will always be alone and unsupported. Additionally, catastrophizing is evident as they anticipate the worst outcomes, feeling that their situation is inescapable and will only get worse. The fear of being misunderstood or judged further isolates them, exacerbating their sense of peril and the constant threat of their symptoms overwhelming them.

Thirdly, the Depressed Self is also navigated through status loss cues. The status loss cues articulate struggles with identity construction and symptoms like difficulty sleeping and loss of interest, highlighting depression’s pervasive impact. Cognitive biases such as catastrophizing play a significant role in shaping these perceptions. For example, one individual expressed, “***After suffering from severe depression, I experienced insomnia, reluctance to leave the house, loss of interest in everything, relentless self-criticism, decreased memory, impaired judgment, slow reaction, severely affecting my normal life and work***.” These descriptions highlight how depression leads to significant deterioration in one’s self-perception and social standing. Catastrophizing is evident as individuals anticipate the worst possible outcomes for their future, believing that their condition will only continue to deteriorate and permanently affect their ability to function normally. This cognitive bias amplifies the perceived severity of their symptoms, making them feel that recovery is unattainable.

#### The optimistic self

The Optimistic Self is primarily formed by hope cues, which acknowledge progress and express hope for the future, inspiring solidarity within the community. Cognitive restructuring helps individuals challenge traditional depressive narratives and foster moments of positivity. For example, one posted, “***I hope everyone will be fine. I have been persisting all along. After being sick for so long, I have gotten used to it. I have a good attitude. Let us keep going***.” These statements reflect a conscious effort to recognize progress and maintain a positive attitude despite ongoing challenges. By actively engaging in cognitive restructuring, individuals counteract biases like negative filtering and catastrophizing, allowing them to appreciate their achievements and foster a sense of hope. Furthermore, the optimism shared in these posts provides encouragement and a sense of belonging to others facing similar struggles, as expressing hope inspires individuals within the community and fosters a sense of solidarity and collective strength.

#### The resilient self

The Resilient Self is negotiated through resilience cues. Resilience cues show people how to confront inner turmoil, seek professional help, and engage in self-care practices like exercise and pursuing personal interests. For example, an individual shared, “***Afterwards, it’s about cooperating well with treatment, exercising more, which may help me restrain those negative thoughts and make myself more positive and optimistic***.” These posts demonstrate a proactive approach to coping with depression, emphasizing personal agency and effort. Engaging in activities like exercise, hobbies, and therapy are common themes in resilient self-presentations, illustrating how individuals confront cognitive biases head-on to manage their symptoms and improve their overall well-being.

Additionally, the Resilient Self is reinforced by advocacy cues, which involve strategic actions aimed at promoting awareness, influencing policy, and combating stigma and discrimination related to mental health. For example, one post stated, “***I share my journey with depression online to help others understand that it’s okay to seek help and to push for better mental health resources***.” These posts challenge negative self-perceptions and societal stigma by advocating for acceptance and understanding of mental health issues. Through advocacy cues, individuals reframe depression in a more positive light, emphasizing growth, self-awareness, and the strength gained from overcoming challenges. These narratives empower individuals by validating their experiences and promoting a positive self-concept.

## Discussion

This research explores self-presentation posts about depression on social media, addressing the gaps in existing literature regarding how individuals with depression present themselves online, navigate stigma, and construct their identities. The findings from this study contribute significantly to the field of health communication by revealing the intricate ways in which individuals with depression use social media to manage and express their mental health experiences.

Firstly, one of the key contributions of this study is the identification of nuanced themes in self-presentation posts, which extend beyond those typically discussed in the literature. Previous studies have primarily highlighted themes such as social challenges, coping strategies, and peer support (34–36), focusing on the “what” and “how-to” aspects of depression experiences. In contrast, this study uncovers not only the struggles and coping methods but also a deeper layer of self-awareness and emotional experience. Specifically, the themes of self-awareness and emotion management, which include making sense of one’s mental states and emotional navigation, highlight the reflective practices individuals engage in to understand and manage their emotions. This suggests that individuals can utilize social media to elicit external support and facilitate internal cognitive processing. Specifically, through engagement on social platforms, individuals with mental disorders can particularly overcome negative cognitive biases (e.g., negative filtering) and achieve more positive cognitive restructuring. This important finding theoretically contributes to the understanding of mental health communication by emphasizing the dual role of social media as a space for seeking help and engaging in self-reflection.

Secondly, the analysis of stigma and challenge cues in this study further enriches the literature by demonstrating the complex interplay of these elements within individual posts. The prevalence of stigma cues such as status loss and peril, as revealed in this study, underscores the pervasive influence of cognitive biases on individuals with mental disorders. Negative filtering and overgeneralization, as discussed by Corrigan et al. (18) and Wood et al. (21), manifest in users focusing disproportionately on negative aspects of their experiences. This selective attention not only amplifies feelings of vulnerability and diminished self-worth but also influences how these individuals present their narratives on social media. Conversely, the emergence of challenge cues in later parts of posts illustrates a shift toward resilience and optimism (22). This transition mirrors cognitive restructuring, suggesting that even amidst pervasive stigma, there is a cognitive recalibration toward hope and recovery. This dynamic interaction between stigma and challenge cues further highlights social media’s dual role: while it can perpetuate stigma, it also serves as a transformative space where individuals can assert control over their narratives, fostering empowerment and encouraging a re-engagement with society.

However, our study also reveals a divergence from Li et al. (9) who noted a predominance of challenge over stigma cues. Such a divergence is possibly due to their broader thematic scope that included personal experiences, self-help strategies, and medical advice, fostering more positive expressions. In contrast, our focus was on posts detailing the psychological and physical burdens of depression, such as feelings of helplessness, isolation, and side effects from medication, which typically yield more negative indicators. Besides, the anonymity provided by the platform RED may also contribute to the prevalence of these negative cues, as users can express raw emotions without disclosing their identity, unlike on identifiable platforms (e.g., Youtube) where content tends to be more cautiously phrased. In addition to differences, the current results also align with previous research [e.g., (38)]. Specifically, our study likewise noted fewer peril cues, such as discussions on suicide, compared to status loss cues like psychological discomforts. This trend may be influenced by platform algorithms that censor overly negative content to mitigate the incitement of negative emotions (34). Such algorithmic moderation in the end not only diminishes the visibility of negative content but may also shift the overall emotional tone of posts toward positivity (e.g., specially the expressions of relief and hope at the end of the posts).

Thirdly, the study’s exploration of identity construction through self-presentation posts reveals the dynamic nature of self-presentation in the context of depression. The identities of Depressed Self, Optimistic Self, and Resilient Self are constructed through the interplay of stigma and challenge cues, reflecting the influence of cognitive biases and the potential for cognitive reframing. The Depressed Self, characterized by negative attribution, peril, and status loss cues, illustrates the pervasive impact of cognitive distortions (such as negative filtering, overgeneralization, and catastrophizing) on self-perception (29, 30). In contrast, the optimistic and resilient selves, shaped by hope and advocacy cues, highlight the capacity for self-empowerment and resilience and the positive thrusts of cognitive restructuring. This theoretical contribution deepens the understanding of narrative identity construction in mental health communication, showing how individuals with depression negotiate their identities amidst stigma and support.

**Figure 2:**
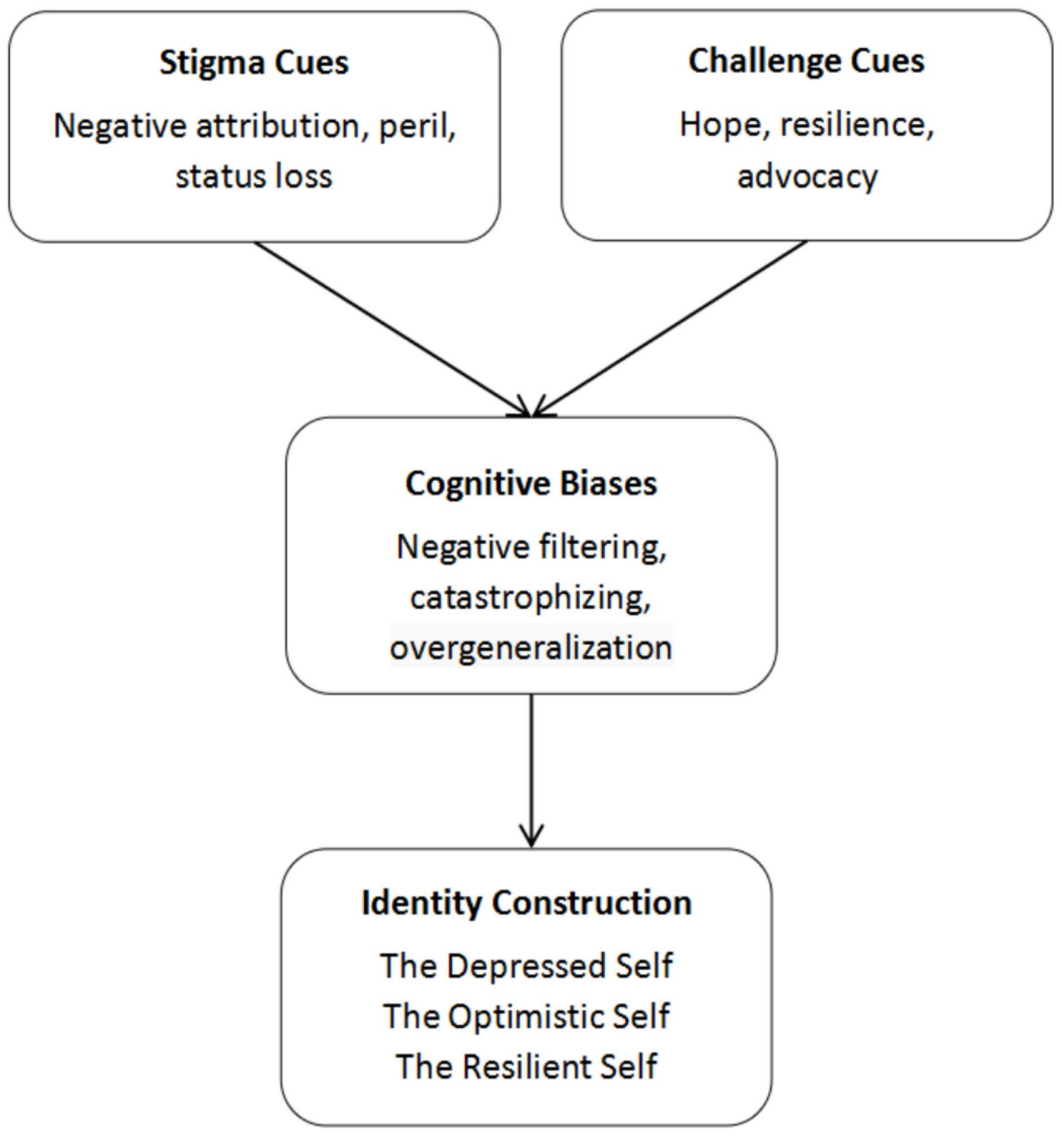
Pathways from depression-related cues to identity construction via cognitive biases on social media.

The present study has then proposed a conceptual model (Figure 2) which outlines the interpretive process through which individuals construct identities in the context of depression-related discourse online. This model begins with the presence of stigma cues (e.g., expressions of shame and social rejection) and challenge cues (e.g., hope, resilience, and advocacy) in users’ narratives. These cues are filtered through cognitive biases, such as negative filtering, overgeneralization, or catastrophizing, which shape how individuals interpret and internalize their experiences. The culmination of this process is the construction of distinct identity types, including the Depressed Self, the Optimistic Self, and the Resilient Self.

Finally, this study also has practical implications. The research demonstrates the transformative role of social media in mental health advocacy and intervention. Insights from Xiaohongshu show that individuals openly discuss their depression, utilizing the platform’s anonymity and real-time feedback. These discussions offer mental health professionals valuable data on common cognitive biases and challenges, enabling them to design targeted interventions that reinforce positive narratives and address stigma. Effectively leveraging these insights can strengthen support systems and foster a more supportive online environment for mental health discourse. Additionally, while this study does not directly examine health care professionals, it contributes to the literature by offering insights into how individuals with depression articulate their experiences on social media—often revealing thoughts and emotions not easily expressed in clinical settings. These first-person narratives can help health care professionals better understand patients’ lived experiences, inform more empathetic and culturally sensitive communication strategies, and identify gaps between formal mental health services and the types of support users seek online.

## Limitations and future directions

While this study provides meaningful insights into how individuals with depression present themselves on Xiaohongshu, there are some limitations to consider. One limitation relates to the platform’s predominantly young user base. As a result, the findings may reflect the perspectives and experiences of younger individuals more than those of older adults, who may be less active on the platform or may express their mental health experiences differently. Future research could explore platforms that attract a more age-diverse population in order to better represent the full spectrum of user experiences. Another limitation involves the platform’s algorithmic recommendation system, which personalizes content based on user preferences. Although this feature helps users find relevant posts, it may also reinforce dominant patterns of expression and reduce exposure to less common or alternative narratives. This filtering effect can contribute to the prominence of certain themes while limiting narrative diversity. Future studies could address this by comparing multiple platforms or using sampling methods that reduce algorithmic influence.

## Conclusion

This study advances the field of health communication by examining how individuals with depression use social media to express their experiences, navigate stigma, and construct identities, highlighting the role of cognitive biases in shaping online self-presentation. The dual role of social media as both a platform for stigma and empowerment offers practical insights for designing interventions that counteract stigma and promote positive mental health narratives, fostering a supportive and empowering online environment. By bridging gaps in existing literature, this research contributes to a comprehensive understanding of online mental health communication, emphasizing the need for future research to explore longitudinal changes in these narratives and the impact of different social media platforms on mental health.

## Data Availability

The datasets generated and analyzed for this study are available from the corresponding author upon reasonable request.
