# Supplementary material for: Exploring self-presentation posts of people with depression: themes, stigma, and identity construction
Source: Front Public Health. 2025 Jun 10;13:1558197. doi: 10.3389/fpubh.2025.1558197 (PMC12185929; doi:10.3389/fpubh.2025.1558197)
Supplement: Supplementary file 1 [file Table_1.docx]

# Supplementary materials

**Table S1** *Clusters, Chinese keywords, and English translations*

| Clusters | Chinese keywords | English translation |
| --- | --- | --- |
| Mental Health and Treatment  (depicted in red, N = 35) | 不敢, 不能, 事情, 医生, 医院, 可能, 告诉, 复诊, 希望, 建议, 影响, 心理, 心理咨询, 情况, 情绪, 想要, 接受, 时间, 有点, 检查, 治疗, 焦虑, 特别, 状态, 环境, 生病, 睡眠, 精神, 药物, 记录, 诊断, 身体, 过程, 问题, 需要 | dare not, cannot, thing, doctor, hospital, possibly, tell, follow-up appointment, hope, advice, influence, psychological, psychological counseling, condition, emotion, wish, accept, time, a little, examination, treatment, anxiety, particularly, status, environment, sick, sleep, mental, medication, record, diagnosis, body, process, problem, need |
| Life Stress and Emotion Management  (depicted in green, N = 35) | 一直, 不好, 不想, 世界, 严重, 人生, 办法, 压力, 原因, 发现, 发生, 吃药, 坚持, 失眠, 好像, 好好, 家人, 工作, 心情, 想法, 找到, 无法, 更加, 朋友, 正常, 比较, 活着, 焦虑症, 生活, 看看, 终于, 觉得, 越来越, 选择 | continuously, not good, not want, world, severe, life, method, pressure, reason, discover, happen, take medicine, persist, insomnia, seem like, well, family, work, mood, idea, find, unable, more, friend, normal, comparatively, alive, anxiety disorder, living, have a look, finally, feel, increasingly, choice |
| Self-Awareness and Emotional Experience  (depicted in blue, N = 34) | 一定, 不会, 不到, 其实, 出现, 分享, 原来, 反应, 喜欢, 已经, 帮助, 应该, 开心, 必须, 患者, 感受, 感觉, 抑郁, 没有, 理解, 症状, 看到, 真的, 知道, 确诊, 经历, 经常, 自救, 起来, 躯体, 运动, 重度, 非常 | definitely, will not, not until, actually, appear, share, originally, reaction, like, already, help, should, happy, must, patient, feel, feeling, depression, not have, understand, symptom, see, really, know, diagnose, experience, often, self-help, get up, body, exercise, severe, very |
| *Note.* “N” denotes the number of keywords. | | |
